# Transapical transcatheter mitral valve implantation with J-valve in patients with degenerated mitral bioprostheses

**DOI:** 10.1186/s12872-023-03414-5

**Published:** 2023-08-10

**Authors:** Fan He, Jianmao Hong, Bijun Xu, Shiqiang Wang, Huaidong Chen, Ximing Qian

**Affiliations:** Department of Cardiovascular Surgery, Sir Run Run Shaw Hospital, Zhejiang University, East Qingchun Road 3th, Hangzhou, 310020 Zhejiang China

**Keywords:** J-valve, Transcatheter mitral valve implantation, Valve-in-valve, Transapical

## Abstract

**Background:**

Due to the widespread application of bioprosthetic valve in the treatment of mitral valve disease in recent years, the incidence of valve failure has increased significantly, which is facing the need of reoperation. For high-risk patients, transcatheter mitral valve-in-valve placement is increasingly being used as an alternative to surgical reoperation.

**Case presentation:**

Here we report the successful transapical transcatheter mitral valve-in-valve implantations of J-Valves in 3 patients with high risk of mitral bioprostheses failure. All patients were discharged successfully, and the follow-up results were good 30 days after operation without major complication.

**Conclusions:**

For high-risk patients, transcatheter implantation of the J-valve is a feasible solution for the treatment of degenerated mitral bioprostheses.

## Background

**Fig. 1:**
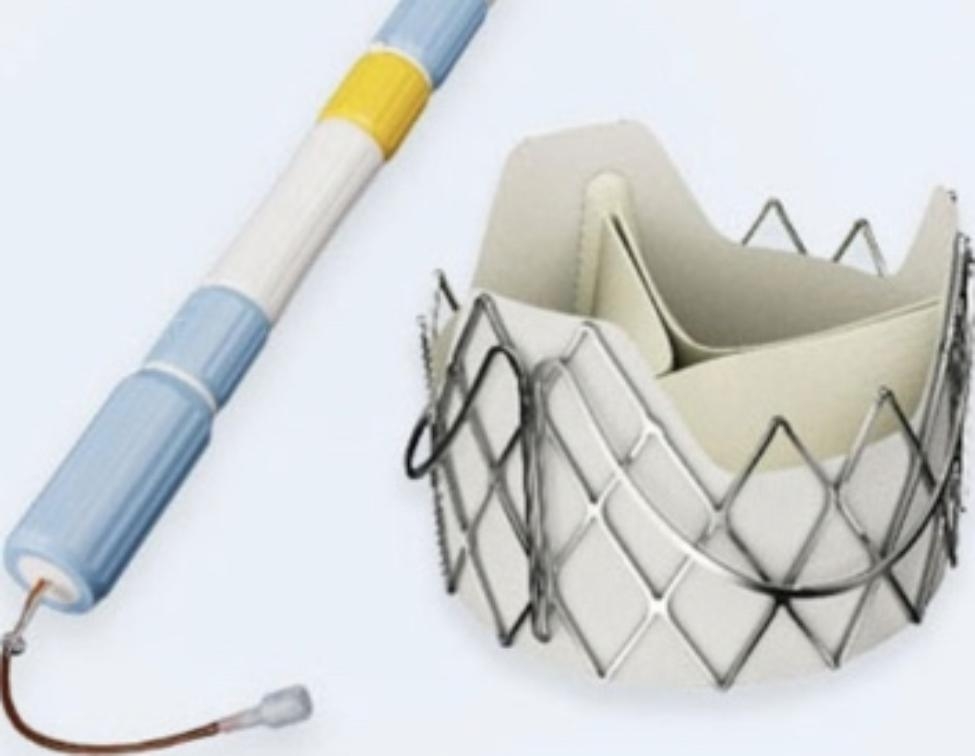
The J-Valve system (JieCheng Medical Technology Corporation Ltd., Suzhou, China)

The use of bioprosthetic valve for the treatment of severe mitral valve disease has continued to increase over the past 20 years due to their excellent properties and the absence of postoperative anticoagulation [1]. However, we are faced with the problem of structural failure of the bioprosthetic valve and the need for reoperation. Studies have shown that 35% of mitral valve replacement patients require reoperation within the first 10 years after surgery [2]. For low-risk patients, Surgical mitral valve replacement (SMVR) remains the standard of treatment. However, for high-risk patients, due to the long operation time, the need for sternotomy again, cardiopulmonary bypass, advanced age and other factors, the perioperative mortality of SMVR is significantly increased [3, 4]. For such high-risk patients, transcatheter mitral valve-in-valve (TMVIV) implantation has emerged as an alternative option [5, 6]. Here, we share our experience of three high-risk patients with degenerated mitral valve bioprostheses who underwent TMVIV via the transapical approach. All the 3 patients were successfully implanted with J-Valve which was made in China (Fig. 1). The perioperative and 30-day follow-up results were good, and the postoperative New York Heart Association (NYHA) class of the 3 patients was greater than or equal to II.

## Case presentation

From October 2021 to July 2022, three patients with bioprosthetic mitral valve failure were admitted to our center and underwent transapical TMVIV using J-Valve. Case One had previously undergone concomitant tricuspid valve repair (TVP) and radiofrequency ablation modified Maze procedure. Case Two had previously undergone concomitant coronary artery bypass grafting (CABG) and MAZE procedures. All three patients had STS scores greater than 8% or logistic EuroSCORE II scores greater than 10%. Preoperative evaluation found no left ventricular thrombosis, infective endocarditis, prosthetic paravalvular leakage, left ventricular outflow tract obstruction, heart tumor, and coronary artery disease requiring CABG. Due to the high risk of surgery, all three patients were considered not suitable for reoperation after multidisciplinary discussion. We decided to perform TMVIV. All patients underwent detailed evaluation including echocardiography, electrocardiogram, cardiac CT, coronary CT and laboratory examination before operation. The preoperative baseline data and preoperative echocardiographic data are shown in Table 1.

**Table 1:** Baseline clinical characteristics

Case	1	2	3
Age	70	72	73
Sex	Female	Female	Male
Height(cm)	160	150	168
Weight(kg)	80	50	73
NYHA Class	III	IV	III
Hypertension	No	Yes	Yes
Diabetes mellitus	No	No	Yes
Pulmonary edema	Yes	Yes	No
Prior CABG	No	Yes	No
Atrial fibrillation	Yes	No	No
STS(%)	12.02	11.51	9
Euro-Score II(%)	22.38	28.59	20.72
Previous procedure	MVR + TVP + MAZE	MVR + CABG + MAZE	MVR
Mechanism of mitral valve failure	Stenosis	Regurgitation	Regurgitation
Duration, years	10	10	11
Peak gradient (mmHg)	37	37	23
Mean transvalvular gradient(mmHg)	15	12.7	6
EOA(cm2)	0.86	2.2	2.53
PASP(mmHg)	44	81	71
LVEF(%)	57	76	62

*NYHA, New York Heart Association; CABG, coronary artery bypass grafting; STS, Society of Thoracic Surgeons ;MVR, Mitral valve replacement; TVP, tricuspid valvuloplasty; EOA, effective orifice area; PASP, pulmonary artery systolic pressure ; LVEF, left ventricular ejection fraction*

**Fig. 2:**
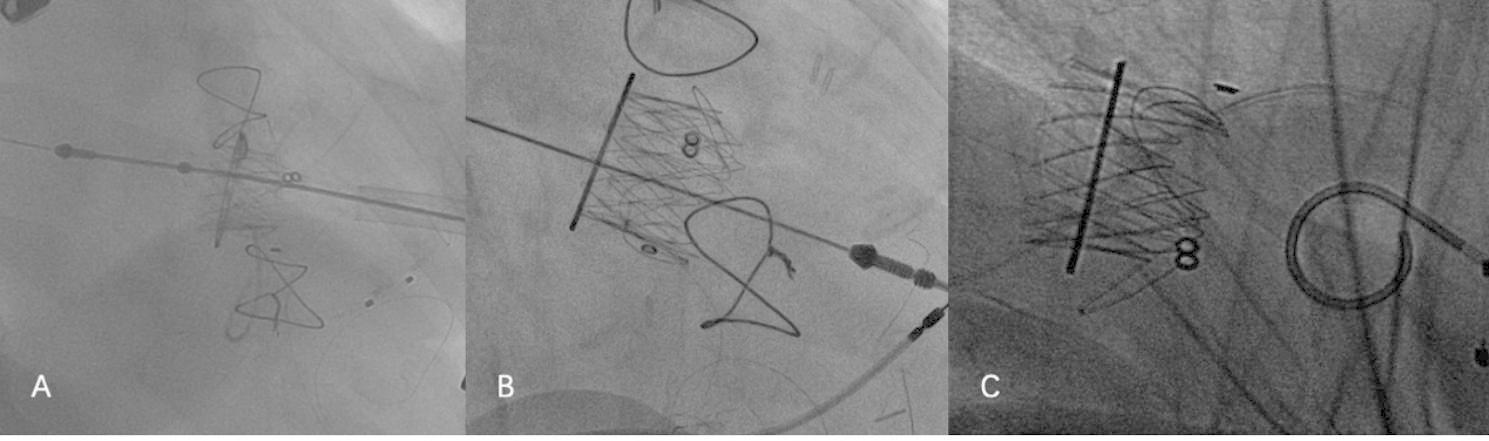
The patients’ intraoperative valve-in-valve implantation of J-valve. **A:** case 1; **B:** case 2; **C:** case 3

**Fig. 3:**
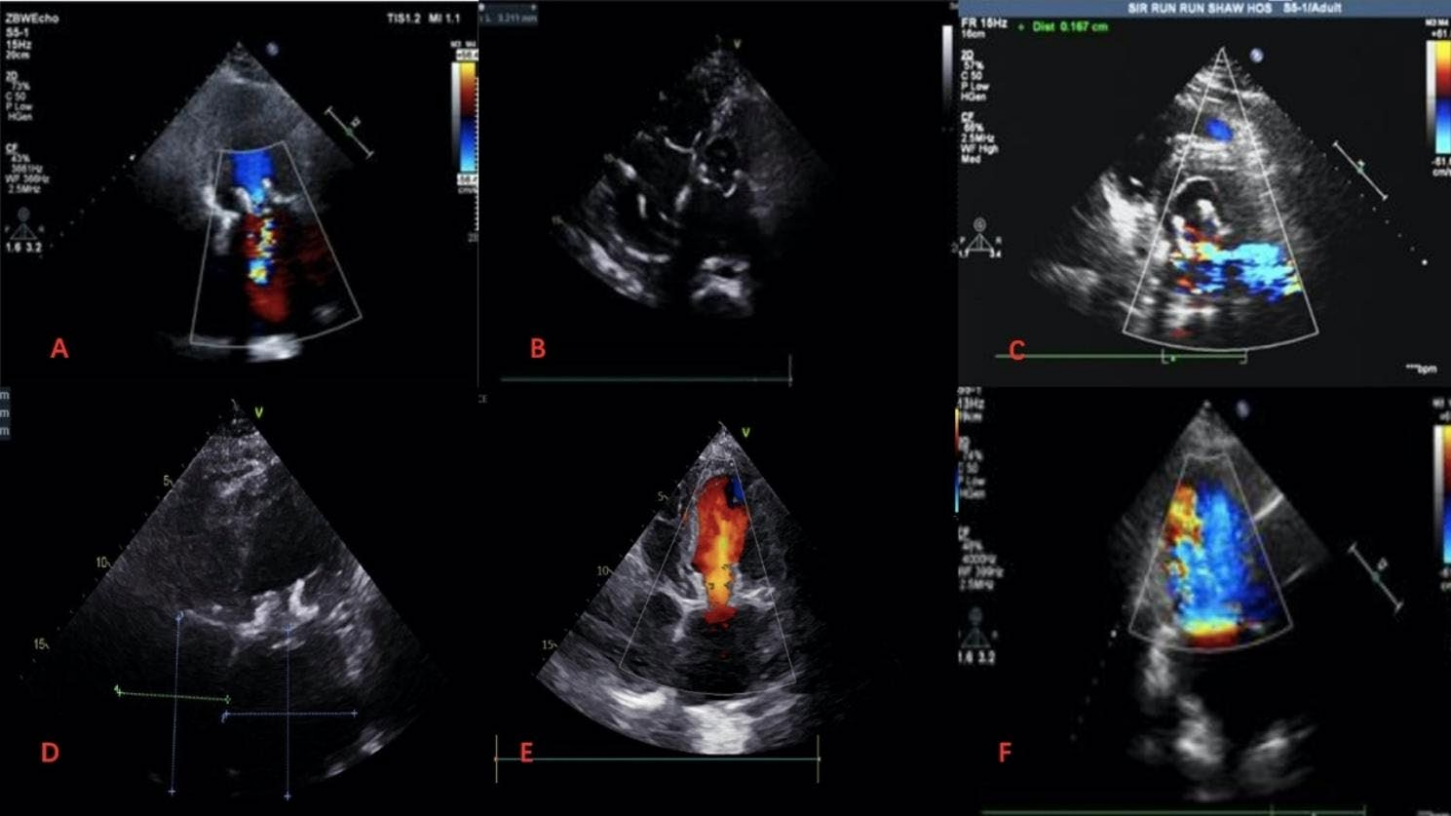
**A**, **B** and **C** were the preoperative echocardiography images of cases 1, 2 and 3, respectively. **D**, **E**, and **F** were the images after J-valve implantation respectively

Transapical approach was used in all cases. Intraoperative X-ray and transesophageal echocardiography were used to determine the position of the cardiac apex, and then a small incision was selected to expose the cardiac apex in the fifth or sixth intercostal space of the left chest. Two purses were closed at the apex of the heart using two 3 − 0 polypropylene sutures with felt pads. The 6 F vascular puncture sheath was implanted at the cardiac apex after heparinization, and the 6 F loach guide wire was exchanged into a pigtail catheter through the orifice of the mitral valve bioprosthesis, and then a superhard guidewire was guided into the left atrium through the pigtail catheter. The pigtail catheter was removed, and the J-Valve operating system was placed into the left ventricle through the guidewire. First, three U-shaped positioning keys were released, and the delivery catheter was moved toward the left atrium to deliver the keys to the three sinus of the prosthetic valve. The J-Valve was then released and fixed to the surgical heart valve (SHV) with the assistance of the positioning keys. Finally, the delivery system was removed, and the valve condition and paravalvular leakage were evaluated by TEE and left ventricular angiography. If a significant paravalvular leak was identified, post-dilatation was performed to transcatheter heart valve (THV) by placing a balloon through a guidewire at a ventricular pacing rate of 180 beats per minute to obtain better hemodynamic and morphological parameters. All the 3 patients were operated successfully. There were no complications such as perivalvular leakage, interventional valve displacement, and conduction block during transesophageal echocardiography and electrocardiogram monitoring. Warfarin was routinely given after operation for 3–6 months, and the international normalized ratio (INR) was maintained between 2 and 3. The 30-day follow-up results of the patients were good. There were no operation-related deaths, perivalvular leakage, conduction block, thromboembolism and bleeding complications. Mitral valve orifice velocity, effective valve orifice area and transvalvular pressure gradient were all improved. The patients’ intraoperative valve-in-valve implantation process were shown in Fig. 2. The surgical data of the patients and the follow-up results at 30 days after surgery were shown in Table 2. The preoperative and postoperative TTE images as Fig. 3.

**Table 2:** Procedural details and 30-day outcomes after operation

Case	1	2	3
Failed prosthesis type	Hancock II	Hancock II	Hancock II
SHV size mm	29	29	29
THV mode	J-valve	J-valve	J-valve
THV size mm	25	25	25
Balloon pre-dilation	No	No	No
Balloon post-dilatation	No	Yes(24#)	Yes(24#)
ventilation time(h)	13	14	8
ICU days	2	2	1
30-day outcomes			
EOA(cm2)	1.83	1.96	1.72
Mitral inflow velocity(m/s)	2.2	2.05	1.77
Mean transvalvular gradient(mmHg)	6.9	5.1	5
NYHA Class	I	II	I
Bleeding complication	No	No	No
Stroke	No	No	No
New complete heart block	No	No	No
Procedure-related death	No	No	No
MR grade > mild	No	No	No
PVL	No	No	No

*SHV, surgical heart valve; THV, transcatheter heart valve; ICU, intensive care unit; PVL, perivalvular leakage*

## Discussion and conclusions

With the widespread application of bioprosthetic valves in mitral valve disease in recent years, more and more patients need reoperation due to bioprosthetic valve dysfunction. For high-risk patients, traditional re-thoracotomy will increase the mortality and morbidity of surgical complications. With the great success of transcatheter aortic valve replacement, TMVIV has now also emerged as an alternative to surgery. At present, the most implanted valve in the world is SAPIEN3 valve, which is the only THV approved for TMVIV, and its effectiveness and safety in the early and mid-term follow-up have been fully confirmed [7]. However, the SAPIEN3 valve was only approved by the China Medical Products Administration in June 2021, so it has not yet been widely used. Currently, the most widely used interventional valve in China is the J-Valve System (JieCheng Medical Technology Corporation Ltd., Suzhou, China). J-Valve prosthetic valve is originally a self-expanding TAVR device approved for both aortic stenosis and aortic regurgitation. Features of the J-Valve system include a trifoliate porcine aortic valve, a self-expanding nitinol stent, three U-shaped anatomically oriented “graspers” for optimal positioning, and a polyester skirt covering the outer surface of the valve stent to minimize the risk of paravalvular leakage [8]. Currently, the J-valve system can also be used for valve-in-valve treatment in some high-risk patients with biological valvular destruction. Yuntao Lu et al. reported 26 patients who underwent TMVIV using J-Valve valve, and the results showed that the success rate of surgery was 96.2%, the all-cause mortality rate of 30-day and 1-year follow-up was 3.8 and 16.0%, respectively, and the stroke rate was 0 and 12.0%, respectively [9]. In contrast, the success rate of the 3 patients reported in our study was 100%, and there were no device-related deaths, thromboembolism, or left ventricular outflow tract obstruction events during the 30-day follow-up. All 3 patients returned to normal life after operation, the symptoms of heart failure were improved, and the NYHA class improved to grade II or above. The mean transvalvular pressure gradient decreased from 11mmHg to 5.7mmHg in 3 patients, and no mild mitral regurgitation, paravalvular leakage and valve displacement occurred. Several factors may explain the high success rate of J-Valve valves. First, the J-Valve valve is equipped with three “U” shaped positioning keys, which can be perfectly anchored to the three leaflets of the biological valve prosthesis, thereby reducing the risk of THV displacement after implantation. Second, the J-Valve valve is a short stent system, which reduces the incidence of left ventricular outflow tract obstruction and left ventricular rupture after implantation compared with other long stent valves.

There are currently two surgical approaches for TMVIV: transapical approach and transfemoral approach. Several studies in the field of TAVR have shown deleterious effects of transapical access, possibly related to inherent myocardial damage and the need for a longer recovery period after left-sided thoracotomy [10]. A recent review comparing two approaches to TMVIV showed no significant differences in mortality or other major events [11]. The J-Valve system is currently implanted via the transapical approach, and the operating system via transfemoral vein approach is still in the clinical trial stage, so we can only choose the transapical approach. However, in our experience, the transapical approach allows better coaxial alignment of the THV with the failed bioprosthetic valve prosthesis. Moreover, its shorter operating distance makes it easier to manipulate the catheter.

During TMVIV, it is crucial to select the appropriate size of the valve. A valve that is too large may affect leaflet motion and lead to central regurgitation or reduced leaflet durability, while a valve that is too small increases the risk of valve migration or paravalvular leakage [12]. Usually we combine the true inner diameter of the SHVs measured by CT with the “Valve in Valve” app according to the manual of the manufacturer. Lu et al. suggested that the choice of J-Valve valve with the same inner diameter as the surgical valve for TVMIV can make the THV leaflet fully expanded, so as to obtain a lower pressure gradient and longer durability [9]. All three of our patients had previously undergone replacement of a 29 mm Hancock II surgical valves with a 24 mm true ID. However, the J-Valve was designed with a size of 21,23,25,27,29 mm, so THVs of 25 mm with oversizing + 1 were chosen in order to obtain a larger opening area. However, for patients with severe stenosis or severe calcification of bioprosthesis, appropriate downsizing should be feasible.

In our experience, transapical TMVIV with the J-Valve system is feasible in patients at high risk for mitral bioprosthesis failure, and the short-term follow-up results are good. Further follow-up results require a larger sample size and longer follow-up time.

## Data Availability

Not applicable.

## Abbreviations

SMVR: Surgical mitral valve replacement
TMVIV: Transcatheter mitral valve-in-valve
NYHA: New York Heart Association
TVP: Tricuspid valve repair
CABG: Coronary artery bypass grafting
CT: Computed tomography
SHV: Surgical heart valve
THV: Transcatheter heart valve
INR: International normalized ratio
